# Effect of capitation payment method on health outcomes, healthcare utilization, and referrals in Ghana

**DOI:** 10.1371/journal.pgph.0002423

**Published:** 2024-06-21

**Authors:** Micheal Kofi Boachie, Eugenia Amporfu

**Affiliations:** 1 SAMRC/Wits Centre for Health Economics and Decision Science – PRICELESS SA, School of Public Health, University of the Witwatersrand, Johannesburg, South Africa; 2 Department of Economics, Kwame Nkrumah University of Science and Technology, PMB, Kumasi, Ashanti Region, Ghana; University of Ghana College of Health Sciences, GHANA

## Abstract

Different provider payment systems generate different incentives for patients, providers, and purchasers. Ghana introduced the National Health Insurance Scheme (NHIS) in 2003 and has made reforms to its provider payment methods to create incentives in providers for cost containment. Starting with the fee for service method, it shifted to the Diagnostic Related Group (DRG) method in 2008 to improve cost containment. In 2012 the NHIS began piloting capitation method of payment which has been suspended since 2017 to allow for thorough review. This study uncovers the association between capitation payment system and patient health outcomes, utilization of healthcare services and referral patterns in Ghana based on data collected between November 2012 and January 2013. Using a cross-sectional data on 500 malaria patients who were enrollees of the NHIS from the two payment plans (i.e., capitation and DRG plan), ordered logit, negative binomial and logit regression results showed that patients under capitation were 11.9% less likely to report better health and had 1.583 fewer visits relative to patients under DRG. In relation to referrals, capitated providers were more likely to refer patients than under DRG plans. In the capitated region, better health outcomes were reported by patients of private health facilities. Capitation in Ghana was associated with under-provision of care, hence decreasing any efficiency gain from the reform. Implementors of capitation need to ensure a good monitoring and evaluation system for adequate provision of quantity and quality of care. Some limitations of this study include the use of cross sectional rather that panel data which follows individuals over time and therefore may be more able to provide definite information about cause-and-effect relationships. It also does not account for events before and after the introduction of any payment method. Overall, this study provides valuable information on the implementation policy for reintroducing capitation.

## Introduction

Healthcare financing is changing dramatically in low- and middle-income countries. Many of these countries have developed and implemented national strategies and policies to achieve universal health coverage (UHC) for their citizens [1]. The goal of UHC is to ensure availability and accessibility of quality health services for those in need regardless of their financial situation [2]. The focus of healthcare financing has therefore shifted from out-of-pocket form of financing to health coverage schemes (e.g., health insurance, which may be social, or private) [2]. However, improperly structured purchasing arrangements under a health insurance may undermine progress towards UHC, due to the incentives to engage in over or under provision of services by providers. This leads to inefficiencies in the health system.

How best to pay healthcare providers for their services, out of the pooled resources, is still a subject of considerable debate due to the mixed responses of the different types of payment systems on providers’ attitude and incentives [3–8]. As a result, provider payment systems are continuously going through reforms, especially in low- and middle-income countries, with the aim of mitigating their negative effects and preserving their positive effects. Many payment reforms have attempted to depart from dependence on the traditional volume driven, i.e., fee-for-service (FFS), method to cost-sharing method. FFS encourages over-provision of services and increases healthcare costs rapidly, as evidenced in Thailand, Taiwan, Korea and several other countries [9]. The Diagnostic Related Group (DRG) method of payment which prices the bundle of services can contain cost more than FFS. However, it creates incentives for unbundling and upcoding which can escalate costs. Purchasers are therefore turning towards various cost-containment arrangements including capitation or a mixture of different payment systems [7,8].

The cost experiences under DRG, among others, perhaps, have given more popularity to capitation as an alternative way, in many reforms, of paying healthcare providers because of its cost saving potential [6,10]. Capitation provides a fixed fee per enrollee for all services required within a fixed time period, usually a year [6,9,11], and may be modified to reflect different risk or population characteristics. By this, capitation provides no direct connection between payments and a provider’s actual healthcare cost of a patient. Under such circumstances, efficient providers may have surpluses to keep, whilst inefficient ones are punished through the trauma of seeking extra funds to cater for patients on their register [11,12]. The capitation payment method aims to improve efficiency, access, and equity without compromising on the quality of care [13–15]. However, a major drawback is the high potential for healthcare providers to underprovide the optimal services needed and hence reduce quality [3,4]. Providers are efficient if they provide the optimal quantity and quality of services under limited resources. Cost reduction may signify a reduction in inputs or resource use but may not be efficient if optimal output or quality is altered.

In many cases, providers under volume driven and fixed fee payment methods of reimbursements differ in the way they care for patients which may affect patient and treatment outcomes. It may also affect patient satisfaction regarding the service received. The reason is that capitation payments, or fixed fees in general, turn providers into risk bearers, making them financially responsible for each enrollee’s cost of care under the contract. Therefore, providers have an incentive to control cost of care per enrollee by either improving on efficiency or engaging in behaviors that may adversely affect treatment decisions and outcomes [4,16–18]. There is a high potential for such healthcare providers to underprovide services and consequently lower their overall healthcare costs. This incentive is even stronger when monitoring of access and service quality is hard, and also when providers are imperfect agents for patients [4]. Indeed, empirical studies suggest that services like laboratory tests are fewer under capitated plans relative to FFS, though clinical outcomes have not been significantly different for the two plans [19].

Provider payment systems are, therefore, crucial in achieving improved access, quality, equity, and above all efficiency in healthcare delivery since each payment mechanism has implications on cost and treatment decisions and outcomes.

The suitability of a specific payment method is country and context specific as it depends a lot on governance and institutional set-up. Nonetheless, incentives generated under various purchasing arrangements in different jurisdictions have similarities, such as over or under provision of services. Many studies agree that services are likely to be underprovided under capitation since the supplier-induced demand for care under fee-for-service would be curtailed, and providers may find it proper to further reduce demand under capitated plans [4,20,21]. If overall health outcomes are not significantly different for capitated and DRG, then patients would not be negatively affected medically [14,17,22,23].

The objective of the current study to investigate the effect of capitation on patient self-reported health outcomes, hospital visits and referral patterns of Ghanaian National Health Insurance enrollees. The study compares differences in health outcomes, referral patterns and healthcare utilization (outpatient visits) under capitation and DRG. It should be noted that under both the DRG and capitation, the payment for medicines remained under the FFS, hence validating comparison between capitation and DRG. The study tests the conventional argument that capitation leads to efficient utilization of resources [3,4,6].

### Provider payments under the Ghanaian national health insurance scheme (NHIS)

Ghana’s NHIS, established in 2003, paid for all healthcare services for its enrollees through fee-for-service arrangements prior to 2007. These payments include consultation fees, drugs, laboratory test and other cost of treatment according to the benefit package of the scheme and the NHIS guidelines [24]. The NHIS-accredited healthcare providers (public, faith-based, and private) submit claims to the National Health Insurance Authority (NHIA), which is the corporate body that runs the NHIS, for reimbursement. The Authority could not bear the rapid escalation of healthcare expenditure due to large claims payment (caused mostly by increased utilization) in its early years [25–27]. According to the NHIA [27], total disbursements of claims payment increased by 367% between 2005 and 2006. The rising claims necessitated reforms in provider payment mechanisms. Thus, in 2007, the provider payment method was reformed to reflect patients’ disease episode, Ghana Diagnostic Related Groupings (G-DRG), to pay for services and arrest the galloping health expenditures [25,26]. Payment for medicines however remained under FFS.

However, not much was achieved in cost saving due to fraud on the part of schemes and providers as claims payments continued to rise [28]. The G-DRG created a situation where almost every patient was diagnosed with higher price disease, and some facilities got reimbursement for no work done [29]. Consequently, claims payment by the NHIA almost quadrupled of its 2007 figure within two years [27,29,30]. Ghana’s experience with DRG is not an isolated case. Countries such as South Korea, Brazil, Thailand, and Taiwan have had similar experiences [9,31], suggesting that the payment mechanism may generate new incentives either on the part of providers or patients.

To control the escalating expenditures and save the NHIS from collapsing, the NHIA introduced a capitation-based payment system (i.e., patient list system), piloted in the Ashanti region, to cater for defined outpatient primary healthcare services [32] with the exception of medicines which were paid under FFS. Under the piloted capitation, providers received a fixed payment per enrollee per month, under the preferred primary provider (PPP) system, irrespective of the enrollee’s utilization of primary healthcare services. Enrollees who needed secondary level healthcare required referral from their PPP which is reimbursed by the G-DRG. The introduction of capitation was expected to improve pricing and reimbursement activities as well as the efficiency of providers [33].

The capitation payment method was piloted in the whole of Ashanti Region but was not without fierce resistance and opposition from medical providers, pressure groups, and politicians as it continued to be a public discussion [34–36]. To register their displeasure, some of the region’s private medical providers initially withdrew their services under the capitation. The providers and opponents argued that the capitation system would jeopardize the quality of healthcare delivery, particularly primary health care. Capitation was, however, suspended in 2017 until further notice to allow for thorough review [37]. Though the technical components of the Ghana’s capitation plan are to check and solve the problems with the payment system, the incentive for providers to alter treatment decisions cannot be ignored.

There have been studies on the impact of capitation under the NHIS on healthcare utilization and cost containment. For example, Andoh-Adjei, Boudewijns, Nsiah-Boatenget al. [38] showed that both healthcare utilization and claims fell after the introduction of capitation in the Ashanti region. Opoku, Nsiah, Opponget al. [39] also showed that capitation led to efficient use of resources. A synthesis of the evidence showed that capitation is efficient as it reduced cost and provided a stable stream of revenue to healthcare providers. Nonetheless, such cost savings reduced the quantity and quality of care, encouraged skimming on inputs, and patient-dumping [5]. Prior to these, Agyei-Baffour, Oppong and Boateng [40] examined the knowledge, perceptions and expectations of capitation payment system enrollees and providers’ perspective. They found that some enrollees’ attitude towards capitation was poor.

It is however not clear from these studies how such a change in cost affected the health of patients. If reduction in utilization leads to deterioration of health, then any cost saving from the reduction of utilization cannot be efficient. The current study does not only include the health outcome of patients in determining the incentives under capitation, but also examines other incentives such as cost shifting through referrals that could also affect efficiency of the payment scheme. Therefore, a thorough examination of the impact of capitation on the efficient use of resources is important to inform policy on the implementation of the payment scheme when it is reintroduced.

## Methods

Our analysis uses malaria patients to proxy all diseases since it is the major cause of morbidity and mortality in Ghana [41]. Indeed, between 2002 and 2017, malaria has consistently been ranked number 1 among the top ten causes of outpatient healthcare utilization. It was also the major cause of admissions in health facilities [42]. Therefore, using malaria as proxy for all diseases under the health insurance is appropriate. Besides comparison of treatment outcomes of one disease is more valid than that of several diseases. Comparing treatment outcomes of several diseases may be invalid because it would be difficult to determine if variation in outcomes is due to differences in the diseases being treated or differences in payment schemes.

### Setting

Data for the study was collected from the Ashanti and Brong Ahafo (which has currently been divided into Bono East, Brong Ahafo, and Ahafo) Regions of Ghana. Since capitation was piloted in the Ashanti region, the Ashanti region served as the intervention region, while Brong Ahafo region served as the control region. Brong Ahafo was selected as the control group due to its similarities with the Ashanti region with regard to the number of enrollees within the study period. Besides, both regions are in the forest belt of the country and have similar malaria incidence [43].

Ashanti Region is centrally located in the middle belt of Ghana and occupies a total land area of 24,389 square kilometers representing 10.2 per cent of Ghana’s total land area. The region’s population is 4,780,380 (with 51.5% females) representing 19.4 per cent of the country’s population [44]. The region is 61 percent urbanized [44]. As of 2012, the region had 548 health facilities which formed about 18% of all the facilities in the country, with the regional population hospital ratio of 8,980 [45]. As of January 2012, providers serving NHIS patients for primary health care services were paid based on capitation method; 37.8 per cent of the region’s population were active members of the NHIS in 2011 [46].

Brong Ahafo Region (BA) covers an area of 39,557 square kilometers with a population of 2,310,983. The region has slightly more females (50.4%) than males [44]. Urban population constitutes 44.5 per cent of the total population of the region. In 2011, about 46 per cent of the region’s population were active members of the NHIS [46]. NHIA accredited providers in the region are paid based on Diagnosis Related Groupings (DRG) methods as of 2008.

In 2007, Ashanti and Brong Ahafo regions recorded 750 450 and 725 057 malaria cases, respectively. This accounted for about 21% and 20% of all reported cases, respectively [41]. These regions also recorded the highest cases in 2016 [41]. Therefore, focusing on malaria in this study is appropriate since it is one of the major causes of morbidity [47].

### Study design

The study used a cross-sectional research design covering the period 2012–2013. Specifically, the collection of the only available data for the study started in November 2012 and ended in January 2013. Data for this period falls within the period of the capitation intervention in Ghana (2012–2017) and the approval for the data collection was provided by the Department of Economics, Kwame Nkrumah University of Science and Technology, Kumasi Ghana, for MPhil Economics dissertation purposes. Besides, studies (e.g., Andoh-Adjei, Boudewijns, Nsiah-Boatenget al. [38] and Opoku, Nsiah, Opponget al. [39]) that examined the impact of Ghana’s capitation on utilization and quality of care used data between 2012 and 2014. The data therefore may appear old relative to the research period, but it contains relevant information that can be analyzed for lessons to be drawn should Ghana reintroduce capitation and/or for other countries that may be considering introducing the payment mechanism.

Structured questionnaires were the main instruments used in eliciting information from enrollees. This cross-sectional design generally allows different variables to be compared simultaneously. Respondents must have used a health facility for a malaria episode at most two months before the survey. The two-month recall period was arrived at after a pilot data collection using a five month recall period. The recall period was reduced when we found that some respondents struggled to recall. At two months, however, it was found that there was no struggle with recall. Thus, two-month recall period was used. The questionnaire contained questions on the type of facility visited and the ownership status of such facilities, whether enrollees paid additional fees aside their health insurance subscription. Respondents were also asked to give the number of visits they have had after the initial visit; and finally, they were asked to rate their health outcomes ranging from very poor to excellent. In addition, information on income, age, employment, and educational levels were collected see appendix for the questionnaire.

The sample size was determined scientifically. Using 5 percent error margin and 95 percent confidence interval and using the NHIS enrollees in the Ashanti and Brong Ahafo regions as the populations of interest, a total of 500 participants were included in the study. The choice of 0.05 error margin and confidence interval are very common in social science studies because they minimize the chances of rejecting H_0_ when it is true. The sample size for Ashanti region was 377 while that of Brong Ahafo was 382. The required sample size was based on Yamane [48] as shown in Eq 1. However, due to time and financial constraints, sample sizes of 250 were used for each region.

$$n=\frac{N}{1+N{\left(e\right)}^{2}}$$ (1)

Where n = the required sample size, *N* is the NHIS member population in each region and e is the error level which is assumed to 5%. Given the smallness of the sample size, vigorous specification tests were run to ensure that there was no specification problem with the results.

To select participants for the study, a starting point was determined by the enumerators by selecting the first house to visit. Afterwards, every tenth house was visited. In the absence of eligible members, the next house was visited. In the house, NHIS enrollees present who had visited a health facility due to malaria within two months before the interview were approached to respond to the questions posed by the enumerator. As indicated earlier, this study uses former malaria patients since malaria has been the major cause of morbidity and mortality in both children and adults in Ghana. Using malaria increases the probability of meeting a potential enrollee for interview hence reducing the time for data collection.

### Outcome measures

This study collected information on the patient—reported health status or health conditions under capitation and DRG plans for those who received outpatient malaria treatment in an NHIS accredited health facility. Thus, self-reported health status is used as a measure for health outcome. The specific questions asked respondents were “in general, how would you rate your health condition: Excellent [] Very Good [] Good [] Poor [] Very Poor []” for health outcomes, “how many visits have you made to your doctor after the initial visit?” for utilization and “were you, at a point, referred to another facility/hospital? Yes [] No []” for referrals.

Other outcomes of interest are referrals and hospital visits. Referrals are basically a switch from capitated to non-capitated service. For this reason, even referral health facilities can refer patients from primary care to secondary care or non-capitated care. For the purposes of this study, enrollees are active members of the NHIS at the time of the survey since such people were eligible to receive healthcare services covered by the NHIS. The study uses cross-sectional data and therefore the analysis focuses on the relationships between the variables under study.

### Inclusivity in global research

Additional information regarding ethical, cultural, and scientific considerations specific to inclusivity in global research is included in the S1 Checklist.

### Statistical analyses

Many factors influence the health of an individual. These include biological and environmental as well as socio-economic factors [49,50]. This study notes that the health status of a person depends on demographic factors (such as education, gender, and age), socio-economic factors (income and education), where he or she receives treatment when sick (i.e., facility information such as type of facility, ownership of the facility), and how the healthcare provider is reimbursed. These factors may also affect healthcare utilization patterns and referrals rates as well as the willingness of patients to switch providers. Income as a variable is measured with error and hence could be endogenous; however, the error diminishes significantly when the period of interest is short. Thus, in this study monthly income is used for the period in which the individual received care. The direct use of income is still a limitation in the study. The result on income is therefore interpreted with caution. To find the association between the provider payment mechanisms and patients health and the behaviors adopted by providers, the following equations are estimated.

$$H_{i}^{*}=\varphi +\beta_{1}X_{i}+\beta_{2}K_{i}+P_{i}+\varepsilon_{i}$$ (2)

$$U_{hi}^{*}=\varphi +\beta_{1}X_{i}+\beta_{2}K_{i}+P_{i}+\varepsilon_{i}$$ (3)

$$R_{i}^{*}=\varphi +\beta_{1}X_{i}+\beta_{2}K_{i}+P_{i}+\varepsilon_{i}$$ (4)

$$S_{i}^{*}=\varphi +\beta_{1}X_{i}+\beta_{2}K_{i}+P_{i}+\varepsilon_{i}$$ (5)

where *X*_*i*_ is a vector of demographic and socio-economic factors (i.e., age, income, and dummy variables representing gender, education, employment status), ***K***_***i***_ is vector of facility information (i.e. facility type and their ownership status), ***P***_***i***_ is a vector of payment methods used in paying healthcare providers (capitation and DRG) affecting the dependent variable and ***ε***_***i***_ is the disturbance term. ***K***_***i***_ and ***P***_***i***_ enter the regression in the form of dummy variables taking the values of 1 and 0. $H_{i}^{*}$ is patient reported health outcome, coded as 0, 1, 2, 3, 4 (very poor, poor, good, very good, excellent, respectively). $U_{hi}^{*}$ is healthcare utilization by the patient measured by the number of visits to the health facility, while $R_{i}^{*}$ represents the referral pattern by providers (coded as 1 for referrals and 0 otherwise). $S_{i}^{*}$ captures the patient’s willingness to continue to receive services from current healthcare providers. Overall, the effect of each variable was captured using dummy variables, except age and income. It should be noted that an important variable that affects health outcome variables, utilization, referrals, and health status, is the severity of illness, which is not included as an independent variable, and hence is captured by the error term, *ε*_*i*_. Besides, the prevalence of the plasmodium falciparum parasites which cause the severest malaria are highest in the two regions, being in the forests belt compared to the other regions in the country. [53] Thus, the regional difference in outcomes cannot be driven by severity of illness but by the main policy difference, provider payment method, capitation.

For the analysis, we used ordered logistic regression to determine the effects of the independent variables on patient self-reported health specified in (2). The choice of ordered logistic regression was based on the ordered nature of the dependent variable. The health outcome (status) variable was ranked from very poor to excellent hence the use of ordered logit in modelling (2). The impact of provider payment method on healthcare use or visits was obtained by estimating (3) with negative binomial estimator due to the count nature of the dependent variable. The negative binomial was chosen rather than Poisson, to avoid the restriction of equality of mean and variance in Poisson. As can be seen in Table 2, the variance for visits exceeds the mean, hence justifying the negative binomial as the appropriate model rather than Poisson. Finally, to examine the effect of the payment method (i.e., capitation) on referrals, we employed binary logistic regression to estimate (3) and (4). Binary logistic regression is appropriate when the dependent variable is dichotomous (i.e., takes the values of 0 or 1). To give intuitive meaning to the results, we report marginal effects. Data analysis was performed using STATA version 11. The choice of STATA software was due to its availability to the authors. The multiple regressions analysis were preceded by bivariate regression analysis to examine the relationship between the dependent variables and the payment system (i.e., capitation or DRG).

In summary, the study used structured questionnaire to collected data between November 2011 and January 2012, using Brong Ahafo region as the control group and Ashanti region as the treatment group, to examine the impact of capitation on selected outcomes: utilization of care, rate of referrals and health outcomes. Whilst the negative binomial multivariate regression was used to examine the effect of capital in utilization, binary logistic regressions were used for the analysis of the impact on referrals and health outcomes.

## Results

### Descriptive Statistics

Majority of the enrollees, 55.20%, are females; 31.2% of the respondents were unemployed as at the time the study. In terms of education, approximately 22% were graduates from tertiary institutions. Thirty-one percent of the respondents (31%) had received basic education, while 29.40% had secondary education and 17.80% have had no formal education. Obviously, the proportion of respondents is higher than the regional proportions for the two regions. However, regression analysis controlled for levels of education to prevent biases in the specification. Their ages ranged between 15 years and 90 years with a mean of 35 years. The mean monthly income was GH¢585.00 (US$292.50). On average, a patient, after initial visit, saw his or her doctor 4.42 times in the two months before the interview. Tables 1 and 2 summarize the characteristics of the sample. The tables provide information on variables such as gender, education and healthcare utilization (Table 1) and income (Table 2).

**Table 1:** Sample characteristics (percentages).

Patient Information/ Variable	All Regions	Ashanti	Brong Ahafo
**Gender**:			
Male	44.80	47.60	42.00
Female	55.20	52.40	58.00
**Level of Education**:			
uneducated	17.80	24.4	11.20
Basic (JHS)	31.00	24.8	37.20
Secondary (SHS)	29.40	23.6	35.20
Tertiary (postsecondary)	21.80	27.2	16.40
**Employment Status**			
Employed Patients	62.20	70.40	66.00
Unemployed Patients	31.80	29.60	34.00
**Ownership status of facilities visited**			
Government	55.20	46.00	64.40
Mission	16.20	18.40	14.00
Private	28.60	35.60	21.60
**Type of facility Visited**			
Teaching/ Regional Hospital	18.40	12.40	24.40
(District) Hospital	17.40	17.60	17.20
Clinic	35.40	35.20	35.60
Health Centre	28.80	34.80	22.80
**Payment of Additional fees aside Insurance**			
Paid Additional Fees	73.80	81.60	66.00
No Additional Fees Paid	26.20	18.40	34.00
**Preferred Primary Care Provider (PPP, Ashanti)**			
Visited Chosen PPP	68.00	68.00	N/A
**Referral Pattern**			
Patients referred	42.20	54.40	30
**Health outcomes/ status**			
Very poor health status	9.00	12.00	6.00
Poor health status	6.60	7.60	5.60
Good health status	20.60	24.40	16.80
Very good health status	32.40	30.80	34.00
Excellent health status	31.40	25.20	37.60

JHS: Junior High School; SHS: Senior High School. See S1 Data for the data.

**Table 2:** Other characteristics.

Variable	Both Regions	Ashanti	Brong Ahafo
	Mean (SD)	Mean (SD)	Mean (SD)
Age (Years)	35.05 (13. 99)	35.75 (13.87)	34.32 (14.10)
Income (GH¢ p. m)	585 (352.09)	675.97 (394.91)	494.04 (275)
Visits	4.42 (3.82)	3.78 (3.90)	5.06 (3.64)

Standard deviation in parentheses. Exchange rate: US$1.00 = GH¢2.00 (average for 2013).

In relation to the type of facilities patients sought treatment and who owns such facilities, most of the respondents (55.20%) reported using publicly owned healthcare facilities, while 16.20% sought treatment from facilities owned and operated by religious bodies usually known as Mission hospitals and/or clinics. Private healthcare providers served 28.60% of the respondents.

### Regression results

#### Effect of payment methods on health outcomes

The bivariate analysis of health outcomes and capitation produced a coefficient of -0.635 (p<0.001) implying a negative relationship between capitation and health outcomes. Presented in Table 3 are the results on the effect of provider-payment mechanism on patient self-reported health status and hospital visits. The table also includes the marginal effects from ordered logistic and negative binomial regressions. Income (β = 0.001; p<0.05), gender (females: β = 0.492; p<0.01), education (secondary: β = 0.852; p<0.01; tertiary: β = 0.640; p<0.01), capitation (β = -0.600; p<0.01) and mission ownership (β = 0.565, p<0.01) were statistically significant in influencing self-reported health outcomes, whereas the effect of employment status, private ownership, and facility type on health status were statistically insignificant at conventional levels.

**Table 3:** Effect of payment system on health status and visits.

	Self-reported Health		Hospital Visits	
VARIABLES	Coefficient (β)	Marginal effects	Coefficients (β)	Marginal Effects
Age	-0.011*	-0.002*	0.010***	0.043***
	(0.006)	(0.001)	(0.003)	(0.011)
Income	0.001**	0.0001**	0.001***	0.002***
	(0.000)	(0.000)	(0.000)	(0.000)
**Gender**				
Ref = Male				
Female	0.492***	0.097***	0.095	0.421
	(0.168)	(0.033)	(0.071)	(0.316)
**Employment status**				
Ref = Unemployed				
Employed	-0.107	-0.021	-0.263***	-1.166***
	(0.191)	(0.038)	(0.077)	(0.347)
**Education**				
Ref = No/less than JHS				
JHS	0.488*	0.096*	-0.086	-0.379
	(0.264)	(0.052)	(0.109)	(0.482)
SHS	0.852***	0.168***	-0.053	-0.236
	(0.277)	(0.054)	(0.111)	(0.493)
Tertiary	0.640**	0.126**	-0.083	-0.367
	(0.286)	(0.056)	(0.116)	(0.516)
**Provider-Payment Method (PPM)**				
Ref = DRG				
Capitation	-0.600***	-0.119***	-0.357***	-1.583***
	(0.178)	(0.035)	(0.075)	(0.343)
**Ownership of Facility**				
Ref = Government				
Mission Facility	0.565**	0.112**	-0.073	-0.322
	(0.260)	(0.051)	(0.104)	(0.463)
Private Facility	-0.090	-0.018	-0.206**	-0.915**
	(0.219)	(0.043)	(0.094)	(0.418)
**Co-payment**				
Ref = No additional fees				
Paid Additional fees	-0.286	-0.057	-0.439***	-1.948***
	(0.202)	(0.040)	(0.082)	(0.378)
**Facility type**				
Ref = Health center				
Teaching hospital	0.256	0.051	-0.048	-0.212
	(0.243)	(0.048)	(0.100)	(0.445)
District hospital	0.189	0.037	0.021	0.094
	(0.227)	(0.045)	(0.091)	(0.405)
Constant			1.631***	
			(0.170)	
Ln Alpha			-1.069***	
			(0.107)	
Pseudo R-sq	0.049		0.034	
Observations	500	500	500	500

Standard errors in parentheses: ** p<0.01, ** p<0.05, * p<0.1.

Of interest is the provider payment method. The coefficient (β = -0.600, p < 0.01) shows that capitation affects health outcomes of patients negatively, holding all other variables constant. The marginal effect of -0.119 (p < 0.01) implies that, holding all other factors constant, a move to capitation, on average, reduces health outcome by about 12 percent. To find out how changes in health outcomes due to capitation varied according facility type, and ownership, capitation was interacted with facility types as well as ownership type and the results, reported in the Appendix, show that only the interaction between capitation and private facility was statistically significant (OR = 3.888, p < 0.01) meaning that the odds of patients of private health facilities in the capitated region reporting excellent health versus good to very poor health is 3.89 times as high as those of public facilities in the capitated region, holding all other factors constant.

#### Provider payment methods and healthcare utilization

The bivariate regression results showed a fall in healthcare utilization in the capitated region with a coefficient of -0.293, (p < 0.001). The rest of the discussion focuses on the multiple regression. With respect to healthcare utilization, age (β = 0.01, p<0.01) and income (β = 0.001, p<0.01) employment status (β = -0.273, p<0.01) were significantly associated with hospital visits (see Table 3). The coefficient of capitation was negative and statistically significant (β = −0.357, p<0.05) meaning that the logs of expected counts of visit in the capitated area is 0.357 less visits than the DRG area. The marginal effect for capitation was -1.583 which means that holding all other factors constant, patients in the capitated area on average made 1.583 fewer visits compared to the DRG area. Results from the interaction of capitation with facility types, reported in the Appendix, show that only the interaction between capitation and private health facility was statistically significant (IRR = 0.781, p<0.05). The results suggest that compared to other facility types (by ownership) in the capitated region, the number of visits to private facilities in the capitated region decreased by a factor of 0.784, holding all other variables constant.

#### Payment method and referrals by providers

The bivariate regression results showed that the coefficient for capitation was 1.023 implying an increase in referrals with capitation. The results of the regression after inclusion of other independent variables are reported in Table 4. On referral of patients to non-capitated services which may be in other health facilities or to same facilities (if higher level (secondary/tertiary) services are provided), paying additional fees, district hospitals, and clinics had expected signs but were insignificant at 5% significance level. However, female (β = 0.828, p<0.01), capitation, (β = 0.785, p<0), mission facility, (β = 0.817, p<0.05), and private facility (β = 0.745, p<0.05) increased the probabilities of referring malaria patients for higher level services. The marginal effect for capitation was 0.141, which implies that holding all other factors constant, the introduction of capitation increased probability of a malaria outpatient being referred to a specialist, or for higher level treatment increased by 14.1 percent. Results from interacting capitation with facility types show that teaching hospitals had an odds ratio of 0.144 with p < 0.05 but the other interactions were not statistically significant. The results imply that teaching hospitals in the capitated region were 86 percent less likely to refer patients for higher level services compared to health centers. To find out how changes in referrals varied across facility types in the capitated region, capitation was interacted with facility type by ownership and as shown in the Appendix, the results showed no statistically significant difference across facility types in the capitated area.

**Table 4:** Effect of payment system on referrals.

VARIABLES	Coefficient	Marginal Effects
Age	0.012	0.002
	(0.008)	(0.001)
Monthly income	-0.000	-0.000
	(0.000)	(0.000)
**Gender**		
Ref = Male		
Female	-0.828***	-0.149***
	(0.222)	(0.038)
**Employment status**		
Ref = unemployed		
employed	0.366	0.066
	(0.246)	(0.044)
**Education**		
Ref = No/less than JHS		
JHS	-0.635*	-0.114*
	(0.333)	(0.059)
SHS	0.171	0.031
	(0.343)	(0.062)
Tertiary	-0.033	-0.006
	(0.354)	(0.064)
**Provider-Payment Method (PPM)**		
Ref = DRG		
Capitation	0.785***	0.141***
	(0.224)	(0.039)
**Ownership of Facility**		
Ref = Government		
Mission	0.817***	0.147***
	(0.309)	(0.054)
Private	0.745***	0.134***
	(0.273)	(0.048)
**Co-payment**		
Ref = No additional fees		
Paid Additional fees	0.925***	0.167***
	(0.269)	(0.046)
**Facility type**		
Ref = Health Centre		
Teaching hospital	-2.525***	-0.455***
	(0.446)	(0.072)
District hospital	0.020	0.004
	(0.263)	(0.047)
Constant	-1.272**	
	(0.534)	
Pseudo R-sq.	0.235	
Observations	500	500

Standard errors in parentheses.

*** p<0.01,

** p<0.05,

* p<0.1.

#### Diagnostic tests

To establish that the regression models are well specified, the link test was executed. The test results (see Table 5) show no misspecification of the estimated models. Similar conclusions were made based on results from estat gof command. These show that models are well fitted.

**Table 5:** Diagnostic test for regressions.

	Model 2	Model 3	Model 4
Hat	1.012*** (0.131)	1.412 (0.872)	0.991*** (0.135)
Hatsq	-0.042 (0.126)	-0.139 (0.292)	-0.009 (0.059)
Hosmer—Lemeshow chi square			9.79

*** significant at 1% level.

## Discussion

The study found that on average visits were 1.583 fewer in capitated region than DRG region, implying that patients in the capitated region on average, made 1.583 less visits to their physicians than those in the DRG region. This was, however, accompanied by poorer health outcomes of patients in the capitated region compared to those under the DRG plan. Specifically, patients under the capitated plan were 11.9% more likely to report very poor health than those in the DRG region. Thus, the reduced number of visits in the capitated region reflects under-provision of services and hence reduced quality of service in the capitated region. The reduced visits may be a way to reduce the financial risk imposed on providers under capitation. Bossman [51] using Ghanaian data between 2010 and 2014 (which covers the same period for capitation as the current study) showed that the cost per outpatient treatment reduced with capitation accompanied by a significant reduction in utilization. The implication is that with capitation, providers had the incentive to use resources efficiently to maintain output at lower cost. However, this study has shown that the reduced utilization may not necessarily be optimal. Any cost savings realized by the health facilities in the capitated region through reduced utilization was overstated. The inadequate monitoring and evaluation system under the capitation [52] to ensure providers complied to the existing protocols might have been the reason for the deterioration of quality of treatment. Kazungu, Barasa, Obadhaet al. [53] provide a review of several empirical literature on provider payments including literature on capitation in Ghana that used data covering the same period as the current study. The review showed that accountability and monitoring mechanisms associated with provider payment system influence provider behavior [53], and in its absence providers may not serve the best interest of patients. Although previous studies use different approaches, i.e., difference-in-difference in Bossman [51] and a systematic review in the case of Kazungu, Barasa, Obadhaet al. [53] as well as qualitative study Obadha, Chuma, Kazunguet al. [1] their findings are consistent with the current study that capitation is associated with reduced services and may change provider behavior in the absence of strong monitoring systems.

The overstatement of savings from reduced utilization is shown in the increased referrals that accompanied the reduced utilization. Patients in the capitated region were 14.1 percent more likely to be referred for higher level services than patients under the DRG region. Thus, part of the savings recorded as gains to the health facilities in the capitated region were actually shifted as cost to the referred facilities or referred departments in the same facilities that provide higher level services. In other words, the health facilities under the capitated plan shifted part of their cost of treatment to other facilities or departments for treatment under DRG plans. Because treatment of referred patients is paid under the DRG plans, the NHIS had to bear the extra cost shifted to other facilities or departments. The resulting cost shifting then represents a significant reduction of gain, if any, to NHIS from the introduction of capitation. This finding is consistent with results from the analyses of NHIA administrative data covering the same period as the current study, which showed that growth in outpatient utilization and claims expenditure slowed in post capitation period and that capitation payment system had a significant negative effect on utilization in the Ashanti region [38]. However, the current study shows that the reduced expenditure must have been due to cost shifting and so may not represent gains from capitation. As has already been discussed, previous studies have shown how the G-DRG has created incentives for upcoding and unbundling by providers. However, the results in the current study show that despite any inefficiency that existed under the G-DRG, there is no clear indication that capitation outperformed the G-DRG in expenditure reduction and quality improvement.

The study also showed that among the health facility types, according to ownership, patients from private health facilities are 3.89 times as likely to report higher health outcomes as those in mission and public health facilities in the capitated region. This implies that private facilities provided the best quality of care among the facility types (by ownership) in the capitated region. There was no statistically significant difference in reduced visits, in the capitated region, among the health facility types by levels. Given that capitation requires NHIS clients to select their preferred provider, private health facilities must have used quality of care to attract and retain patients. It has been shown that facility features like shorter waiting time, availability of drugs and qualified doctors influence the choice of primary care provider [54]. Our result confirms the recommendation made in Andoh-Adjei, Cornelissen, Asanteet al. [34] that competition among the facility types that exists in Thailand is needed in the Ghanaian system to motivate mission and public health facilities to improve their quality to the level of the private facility.

The study found no statistically significant difference in referrals among the facility types in the capitated region, implying that the increased referrals was statistically the same among the facility types be it public, mission, or private. Given that reduction in visits was also statistically the same among facility types, it follows that private facilities managed resources better than the mission and public facilities to obtain the highest quality outcome. It can therefore be deduced that private health facilities performed most efficiently compared to public and mission facilities in the capitated region.

The health facilities in Ghana are categorized according to the level of care they can provide, with the lowest being Health Centre, followed by District level hospital, and Teaching hospital (tertiary). The results in the study showed that there was no statistically significant difference in the reduction in the number of visits or quality of care across the health facility types, in terms of level of care, in the capitated region. This implies that variation in the quality of care across facilities as a result of capitation, was driven by ownership rather than capacity of the facilities. The results on referrals show that Teaching hospitals in the capitated region were the least likely to refer patients for higher level services. To the extent that referral is used to shift cost to the NHIS through other facilities or departments, teaching hospitals were the least likely to engage in such activity and hence the most efficient. This is expected as the teaching hospital in the data is the last referral health facility in the two regions.

## Conclusion and policy implications

We investigated the association between capitation and healthcare utilization and provider behavior in the Ghanaian health system, using a cross-sectional data collected on patients. After applying logistic, ordered logistic and negative binomial regression techniques to the data, we found that capitation is significantly associated with healthcare utilization, referral rates, and patient self-reported health status negatively. Because capitation imposes a financial risk on healthcare providers, they are more likely to reduce utilization and quality of treatment which in turn affects patient health outcome negatively. The reduced utilization accompanied by the increased referrals could imply cost shifting hence reducing any expenditure gain from the reduced utilization. Private facilities were able to provide the best quality of care compared to public and mission health facilities. The variation of quality of care was driven by facility ownership rather than facility capacity.

The findings provide an impetus for the policymakers, particularly purchasers, to develop capitation, should it be reintroduced, to have an inbuilt monitoring and evaluation mechanisms to mitigate their negative effects. The result from the study that the impact of capitation is driven by facility ownership justifies the need to purchase healthcare services strategically, and monitoring is an important purchasing strategy. By this, policymakers should restructure the capitation payment method as well as other payment methods to prevent under-provision and also monitor and evaluate providers routinely to improve quality of care.

## Limitations

This study has limitations. It does not follow individuals over time and therefore it may not provide definite information about cause-and-effect relationships. The study did not account for events before and after the introduction of any payment method. Additionally, the regressions do not account for facility resources such as the number of beds, wards, medical personnel (e.g., nurses, medical doctors, and pharmacists) and their qualifications, and laboratory equipment due to the difficulty in obtaining such information despite their likelihood of affecting health outcomes. Also, we did not use theoretical model to design our data collection. Since the study uses cross-sectional data, we were unable to conduct cause and effect analysis hence interpretation of the findings should keep in mind of this limitation.

### Future research

We suggest that future research investigating the effects of PPMs focus on causal relationships between provider payment methods and health, and patient and provider behaviors. Further, research on institutional challenges and provider characteristics that influence referrals will provide insights to the referral decisions. Future studies should also consider establishing through qualitative studies why the referrals or utilization under capitation differed.

## Supporting information

S1 Checklist (DOCX)

S1 Appendix (DOCX)

S1 Data (DTA)
