# Supplementary material for: Effect of capitation payment method on health outcomes, healthcare utilization, and referrals in Ghana
Source: PLOS Glob Public Health. 2024 Jun 21;4(6):e0002423. doi: 10.1371/journal.pgph.0002423 (PMC11192346; doi:10.1371/journal.pgph.0002423)
Supplement: S1 Appendix — (DOCX) [file pgph.0002423.s002.docx]

**Appendix**

Table A1: Models with interactions

|  | 1 | 2 | 3 |
| --- | --- | --- | --- |
| VARIABLES | Health Outcomes | Visits | Referrals |
|  | Odds ratios | IRR | Odds ratios |
| Age | 0.989* | 1.010*** | 1.015* |
|  | (0.006) | (0.002) | (0.009) |
| Income | 1.001* | 1.000*** | 1.000 |
|  | (0.000) | (0.000) | (0.000) |
| **Gender** |  |  |  |
| Ref= Male |  |  |  |
| Female | 1.656*** | 1.127* | 0.396*** |
|  | (0.281) | (0.079) | (0.092) |
| **Employment status** |  |  |  |
| Ref = unemployed |  |  |  |
| employed | 0.893 | 0.755*** | 1.471 |
|  | (0.173) | (0.058) | (0.378) |
| **Education** |  |  |  |
| Ref = None/less than JHS |  |  |  |
| JHS | 1.657* | 0.958 | 0.590 |
|  | (0.443) | (0.102) | (0.205) |
| SHS | 2.362*** | 0.927 | 1.265 |
|  | (0.660) | (0.100) | (0.452) |
| Tertiary | 1.975** | 0.908 | 1.051 |
|  | (0.569) | (0.103) | (0.392) |
| **Provider payment method (PPM)** |  |  |  |
| Ref = FFS/DRGs |  |  |  |
| Capitation | 0.513 | 1.536** | 2.044 |
|  | (0.266) | (0.321) | (1.422) |
| **Ownership type** |  |  |  |
| Ref = Government |  |  |  |
| Mission | 2.234** | 0.982 | 1.828 |
|  | (0.897) | (0.142) | (0.857) |
| Private | 0.452** | 0.935 | 3.743*** |
|  | (0.145) | (0.116) | (1.502) |
|  |  |  |  |
| **Facility type** |  |  |  |
| Ref = health centre |  |  |  |
| Clinic | 1.653 | 1.276* | 1.631 |
|  | (0.563) | (0.172) | (0.675) |
| District Hospital | 1.051 | 1.136 | 1.052 |
|  | (0.403) | (0.168) | (0.505) |
| Teaching Hospital | 1.274 | 0.990 | 0.217** |
|  | (0.474) | (0.149) | (0.131) |
| **Co-payment** |  |  |  |
| Ref = No additional fees |  |  |  |
| Additional fees | 0.991 | 0.937 | 1.332 |
|  | (0.268) | (0.099) | (0.517) |
| **Interactions** |  |  |  |
| Capitation*Mission | 0.840 | 0.964 | 1.709 |
|  | (0.453) | (0.200) | (1.132) |
| Capitation*Private | 3.890*** | 0.800** | 0.397* |
|  | (1.731) | (0.146) | (0.220) |
| Capitation*Additional fees | 0.539 | 0.442*** | 2.555* |
|  | (0.218) | (0.069) | (1.422) |
| Capitation*Teaching hospital | 1.727 | 1.141 | 0.134** |
|  | (0.964) | (0.262) | (0.137) |
| Capitation*District hospital | 2.059 | 0.906 | 1.252 |
|  | (1.058) | (0.186) | (0.798) |
| Capitation*Health Centre | 0.759 | 0.708* | 0.568 |
|  | (0.341) | (0.133) | (0.317) |
| **Patient type** |  |  |  |
| Ref = outpatient |  |  |  |
| Inpatients | 0.897 | 1.005 | 2.240*** |
|  | (0.159) | (0.072) | (0.542) |
| Constant |  | 3.223*** | 0.168*** |
|  |  | (0.655) | (0.115) |
| Ln alpha |  | 0.301*** |  |
|  |  | (0.034) |  |
| Pseudo R-sq | 0.0556 | 0.0499 | 0.257 |
| Observations | 500 | 500 | 500 |

Standard errors in parentheses

*** p<0.01, ** p<0.05, * p<0.1
